# A new palladium complex Schiff-base on functionalized nanoboehmite as a reusable and practical catalyst for selective Suzuki C–C bond formation†

**DOI:** 10.1039/d5na00362h

**Published:** 2025-06-17

**Authors:** Samaneh Heydarian, Bahman Tahmasbi, Mitra Darabi

**Affiliations:** a Department of Chemistry, Dez.C., Islamic Azad University Dezful Iran Heydarian@iau.ac.ir; b Department of Chemistry, Faculty of Science, Ilam University P. O. Box Ilam 69315516 Iran b.tahmasbi@ilam.ac.ir

## Abstract

The surface of boehmite nanoparticles (γ-AlOOH NPs) consists of hydroxy groups that enable their surface modification and functionalization. Based on this fact, we first functionalized the AlOOH NP surface with a Schiff-base ligand in this work. The Schiff-base ligand was synthesized from the reaction of *o*-formylphenol and (3,4-diaminophenyl)(phenyl)methanone. Then, palladium nanoparticles were immobilized on it, denoted as Pd@boehmite. Next, Pd@boehmite was investigated using TGA, DSC, SEM, TEM, and BET instrumental methods. Then, Pd@boehmite was used as a powerful catalyst for carbon–carbon bond formation in the Suzuki coupling reaction. Various aryl halide and aryl boronic acid derivatives were investigated using the Pd@boehmite nanocatalyst and all biphenyl products were obtained with high yield and rapid reaction rate. Pd@boehmite showed good selectivity in synthesising biphenyls, when diaryl halide was used. Finally, the recyclability of Pd@boehmite was also examined, and this catalyst showed good reusability.

## Abstract 2

The surface of boehmite nanoparticles was functionalized with a Schiff-base ligand. Then, palladium nanoparticles were immobilized on it (Pd@boehmite). Next, Pd@boehmite was used as a powerful catalyst in the Suzuki coupling reaction.

## Graphical abstract

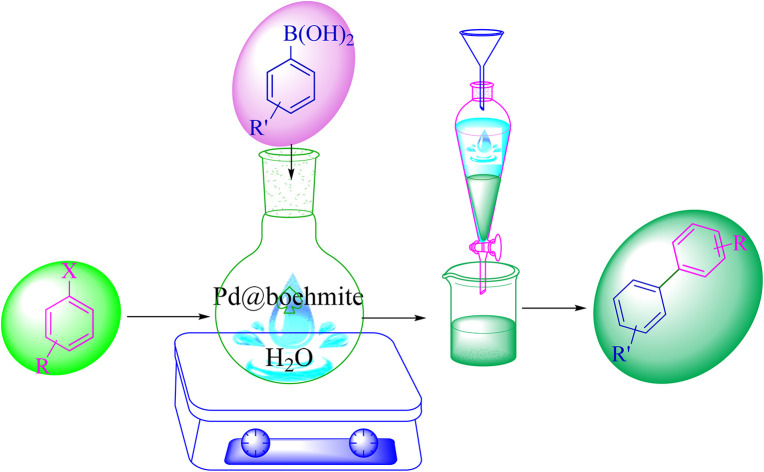

## 1. Introduction

Over the past several decades, the immobilization of homogenous catalysts on inorganic supports has received much attention due to the use of sustainable green chemistry as an appropriate approach to improve their stability, reactivity, and selectivity.^1–3^ Homogeneous catalysts exhibit higher catalytic activity and selectivity than their heterogeneous counterparts due to their solubility in the reaction medium, which increases the accessibility of the catalytic site to the substrate.^4^ Despite these advantages, homogeneous catalysts often face the problem of catalyst separation and product contamination.^5^ Heterogeneous catalysts have been used as recoverable and reusable catalysts in organic functional group transformations.^6^ A variety of approaches have been used to develop heterogeneous catalysts, the general method of which involves the immobilization of homogeneous catalysts on nano-materials as heterogeneous supports.^7–11^ Therefore, to combine the advantages of both catalysts, nano-catalysts emerged as a bridging gap between homogeneous and heterogeneous catalysts.^12,13^ Mesoporous materials, biochar, carbon nanotubes, ionic liquids, boehmite (γ-AlOOH), *etc.* are inorganic and organic supports, which are used to prepare nanocatalysts.^14–20^ However, some of the previous heterogeneous supports such as mesoporous materials require high temperatures for calcination, and the preparation of some catalysts with carbon nanotube and ionic liquid supports is not cost-effective. Also, the preparation of some other heterogeneous catalysts requires a lot of time and exhausting conditions for separation.^13^ In recent years, γ-AlOOH NPs (aluminium hydroxide) have attracted a lot of attention as an insoluble support for the production of heterogeneous catalysts due to their nanoscale dimensions and morphological characteristics, large surface area, narrow pore-size distribution, large pore-volume, and acid–base properties.^17,21,22^ γ-AlOOH NPs are an aluminium oxide hydroxide (boehmite, γ-AlOOH) mineral that contains surface hydroxy groups.^22^ These hydroxy groups give the surface high reactivity and provide a hydrophilic environment that improves the homogeneity of the environment.^17,23^ Also, γ-AlOOH is an inexpensive material and due to its high thermal and chemical stability it is widely used in industries as an absorbent, abrasive, and flame retardant and for filtration or separation, preparing advanced catalysts, and preparing alumina and alumina-derived ceramics. Because of these unique properties mentioned, γ-AlOOH is a promising inorganic insoluble support.^24–27^ Recently, after modification of γ-AlOOH with appropriate ligands to anchor transition metal cations in the structural network of these materials, it can be proposed as a method to prepare new nanocatalysts with active centers, which used the catalytic activity of heterogeneous catalysts in various organic reactions.^28–32^

In this paper, we decided to use palladium metal on γ-AlOOH and investigated its catalytic activity in the Suzuki–Miyaura coupling reaction.

Palladium-mediated cross-coupling *e.g.* Stille, Suzuki–Miyaura, Sonogashira–Hagihara and Mizorouki–Heck reactions are effective processes for carbon–carbon bond formation in many organic transformations and have attracted the most attention from the synthetic chemistry community.^33,34^ Among the various cross-coupling methods available, Suzuki–Miyaura cross-coupling is the most widely used reaction for C–C bond formation due to its high efficiency in various fields such as medicine, pharmaceuticals, herbicides, advanced materials and cosmetics.^28,35,36^ For example, *o*-tolyl benzonitrile, or OTBN, is an interesting chemical compound used as an advanced drug intermediate, particularly in the synthesis of candesartan, losartan, irbesartan, valsartan, and tasosartan, which are used to treat hypertension and heart failure. OTBN is easily synthesized using a cost-effective and environmentally friendly process from the Suzuki coupling reaction.^37,38^ Also, 4′-chloro-2-nitro-1,1′-biphenyl is prepared by Suzuki coupling of 4-chloroboronic acid and 2-nitrochlorobenzene, which is employed in the production of fungicide boscalid.^37,39^ ABT-963 (a COX-2 inhibitor) and Cl-1034 (an endothelin antagonist) are important pharmaceutical compounds that utilize the Suzuki coupling reaction in at least one step of their synthesis.^39^

## 2. Experimental

### 2.1. Preparation of the Pd@boehmite nanocatalyst

**Fig. 1:**
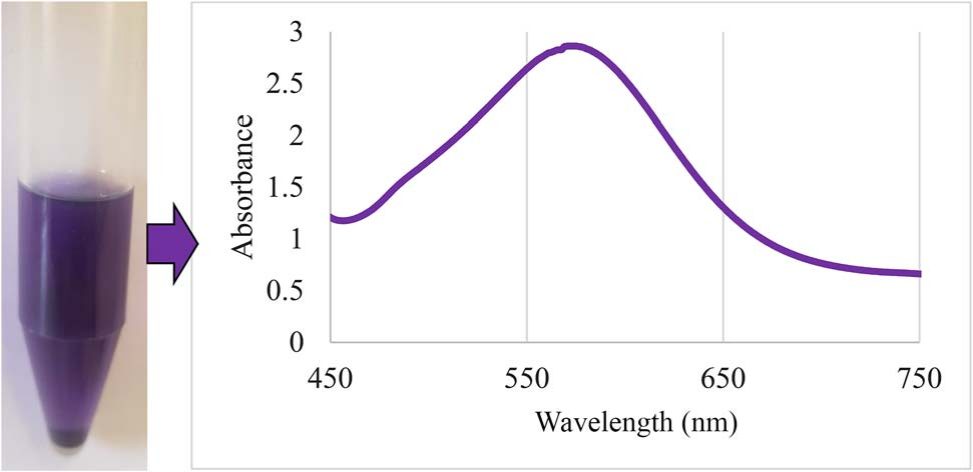
UV spectrum after a ninhydrin test for NH_2_@boehmite.

**Scheme 1:**
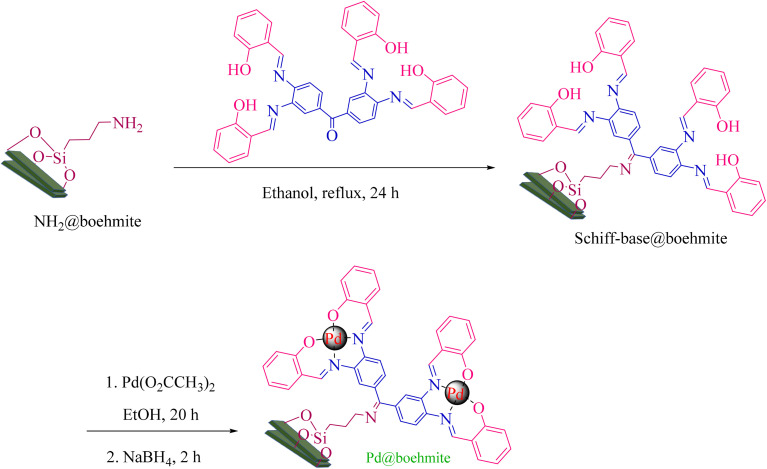
Synthesis of Pd@boehmite.

First, 49.6 g of NaOH was dissolved in 50 mL of distilled H_2_O and added dropwise to 30 mL of Al(NO_3_)_3_·9H_2_O solution (containing 20 g of Al(NO_3_)_3_·9H_2_O). It was stirred vigorously for 20 min. If the reaction mixture solidified during the addition of NaOH solution to Al(NO_3_)_3_ solution, the titration was stopped and the stirring intensity was increased until the reaction mixture became solution again. After the titration, the formed mixture was dispersed in an ultrasonic bath at room temperature for 3 h. Then, the formed gel precipitate was heated at 220 °C for 4 h. The white solid powder obtained was γ-AlOOH NPs (boehmite nanoparticles). The boehmite nanoparticles were washed with distilled H_2_O and dried at 70 °C to remove nitrate impurities. In step 2, 1 g of γ-AlOOH NPs was dispersed in 20 mL of *n*-hexane in an ultrasonic bath for 30 min. Then, 2 mL of (3-aminopropyl)triethoxysilane was added and stirred for 24 h under reflux conditions and a nitrogen atmosphere. The boehmite modified with (3-aminopropyl)triethoxysilane (NH_2_@boehmite) was separated with filter paper, washed 5 times with ethanol, and dried at 50 °C. To prove the successful surface modification of boehmite with (3-aminopropyl)triethoxysilane, a ninhydrin test was performed for NH_2_@boehmite (Fig. 1). The purple color indicates the presence of free amino groups on the boehmite surface, which was identified by a strong peak at 575 nm in the UV spectrum. In step 3, 1 g of NH_2_@boehmite was dispersed in 20 mL of ethanol in an ultrasonic bath and 2 mmol of Schiff-base ligand was added to it. The formed mixture was stirred under reflux conditions for 24 h. The Schiff-base ligand-functionalized boehmite (Schiff-base@boehmite) was separated with filter paper, washed 5 times with hot ethanol, and dried at 50 °C. In step 4, 1 g of functionalized boehmite (Schiff-base@boehmite) was dispersed in 20 mL of EtOH (ethanol) using an ultrasonic bath. 0.5 g (2 mmol) of palladium acetate was added to it and stirred at 80 °C for 20 h. Under the same conditions (without purification), 0.6 mmol of sodium borohydride was added and stirring was allowed for 2 h under the same conditions. The palladium immobilized on boehmite (Pd@boehmite) was separated with filter paper, washed with distilled H_2_O and EtOH, and dried at 50 °C (Scheme 1).

### 2.2. Synthesis of various biphenyl derivatives through the C–C cross-coupling reaction catalyzed by Pd@boehmite

**Scheme 2:**
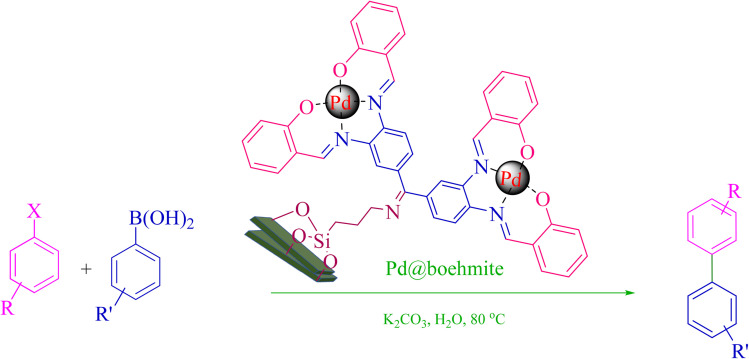
Synthesis of biphenyls *via* the C–C coupling reaction catalyzed with Pd@boehmite.

0.75 mmol of K_2_CO_3_ (0.0517 g), aryl halide (0.5 mmol), distilled H_2_O (1 mL), aryl boronic acid (ABA, 0.5 mmol), and Pd@boehmite (5 mg) were mixed and stirred at 80 °C (Scheme 2). The reaction was controlled with TLC. At the end of the reaction, it was cooled, and Pd@boehmite was removed by paper filtration. It was washed using ethyl acetate. The biphenyl products were extracted from distilled H_2_O using ethyl acetate. The organic solvent was dried using Na_2_SO_4_, and ethyl acetate was evaporated, and biphenyls were obtained.

### 2.3. Selected spectral data

#### 2.3.1 3′,4′-Difluoro-[1,1′-biphenyl]-4-carbonitrile

^1^H NMR (250 MHz, CDCl_3_): *δ*_H_ = 7.74 (d, *J* = 10 Hz, 2H), 7.62 (d, *J* = 10 Hz, 2H), 7.40 (t, *J* = 10 Hz, 1H), 7.30 (m, 1H), 7.26 (m, 1H) ppm.

#### 2.3.2 4-Hydroxybiphenyl

^1^H NMR (250 MHz, CDCl_3_): *δ*_H_ = 7.55 (d, *J* = 10 Hz, 2H), 7.52 (d, *J* = 10 Hz, 2H), 7.42 (t, *J* = 6 Hz, 2H), 7.31 (t, *J* = 6 Hz, 1H), 6.91 (d, *J* = 10 Hz, 2H), 4.26 (br, 1H) ppm.

#### 2.3.3 4-Methoxybiphenyl

^1^H NMR (250 MHz, CDCl_3_): *δ*_H_ = 7.54 (m, 4H), 7.42 (t, *J* = 10 Hz, 2H), 7.25 (m, 1H), 6.98 (d, *J* = 5 Hz, 2H), 3.86 (s, 3H) ppm.

## 3. Results and discussion

### 3.1. Synthesis and identification of Pd@boehmite

First, γ-AlO(OH) NPs were synthesized in an aqueous medium using aluminium nitrate as the Al-source and sodium hydroxide (NaOH) as the base. Then, the γ-AlOOH NP surface was modified and functionalized with a Schiff-base ligand. Finally, the palladium/complex was formed on the immobilized Schiff base ligand (Pd@boehmite). Next, Pd@boehmite was investigated using TGA, DSC, SEM, TEM, and BET instrumental methods.

#### 3.1.1. N_2_ adsorption–desorption isotherms

**Fig. 2:**
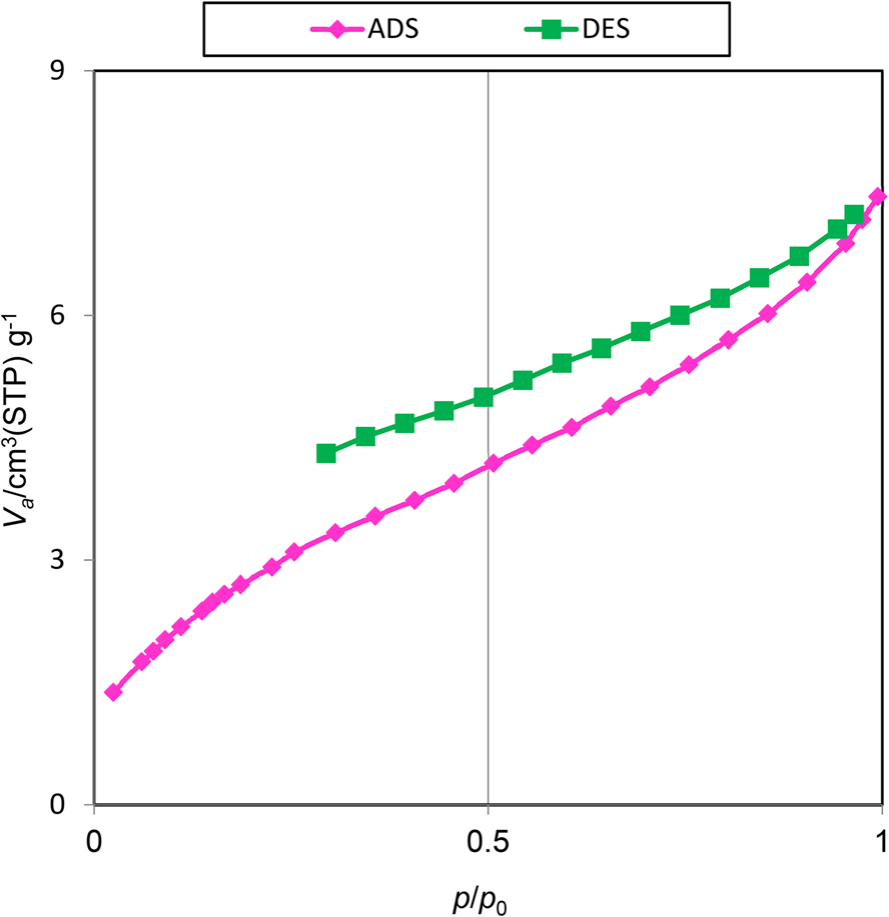
N_2_ adsorption–desorption isotherms of Pd@boehmite.

The surface and pore properties of Pd@boehmite were studied using N_2_ adsorption–desorption analysis. The output results of N_2_ adsorption–desorption analysis are shown in Fig. 2. Based on the BJH and BET analysis from N_2_ adsorption–desorption analysis, the Pd@boehmite surface area is 9.87 m^2^ g^−1^. Also, the pore diameter and pore volume for Pd@boehmite were obtained as 4.64 nm and 0.011 cm^3^ g^−1^, respectively. These resulting diagrams of Pd@boehmite are an IUPAC type I isotherm with an H4 hysteresis loop, which shows slit-like pores (sheet-like),^40^ confirming the expected sheet-like structure for γ-AlOOH NPs.

#### 3.1.2. TGA

**Fig. 3:**
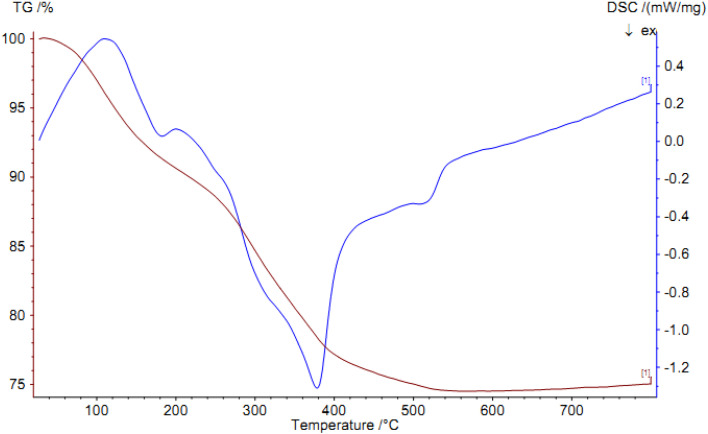
TGA and DSC diagrams of Pd@boehmite.

TGA and DSC diagrams for Pd@boehmite are shown in Fig. 3, which provide useful information about the organic, inorganic, moisture, and other adsorbed solvent content of Pd@boehmite based on weight changes with increasing temperature. As expected, the absorbed moisture and solvents evaporate at low temperatures, which is observed with a weight loss of 8.4% in Fig. 3. Also, the organic content of Pd@boehmite, which includes the propyl chain and the Schiff-base ligand, is observed as a weight loss of 15.3% at 200–450 °C, which is related to the decomposition of the organic part in Pd@boehmite. Because γ-AlOOH is one of the phases of alumina, changes phase to alumina at high temperatures, and has been reported as a starting material for the formation of alumina in many reports,^41–43^ this fact is observed as a weight loss of 1.3% at 450 °C in the TGA diagram.

#### 3.1.3. SEM images

**Fig. 4:**
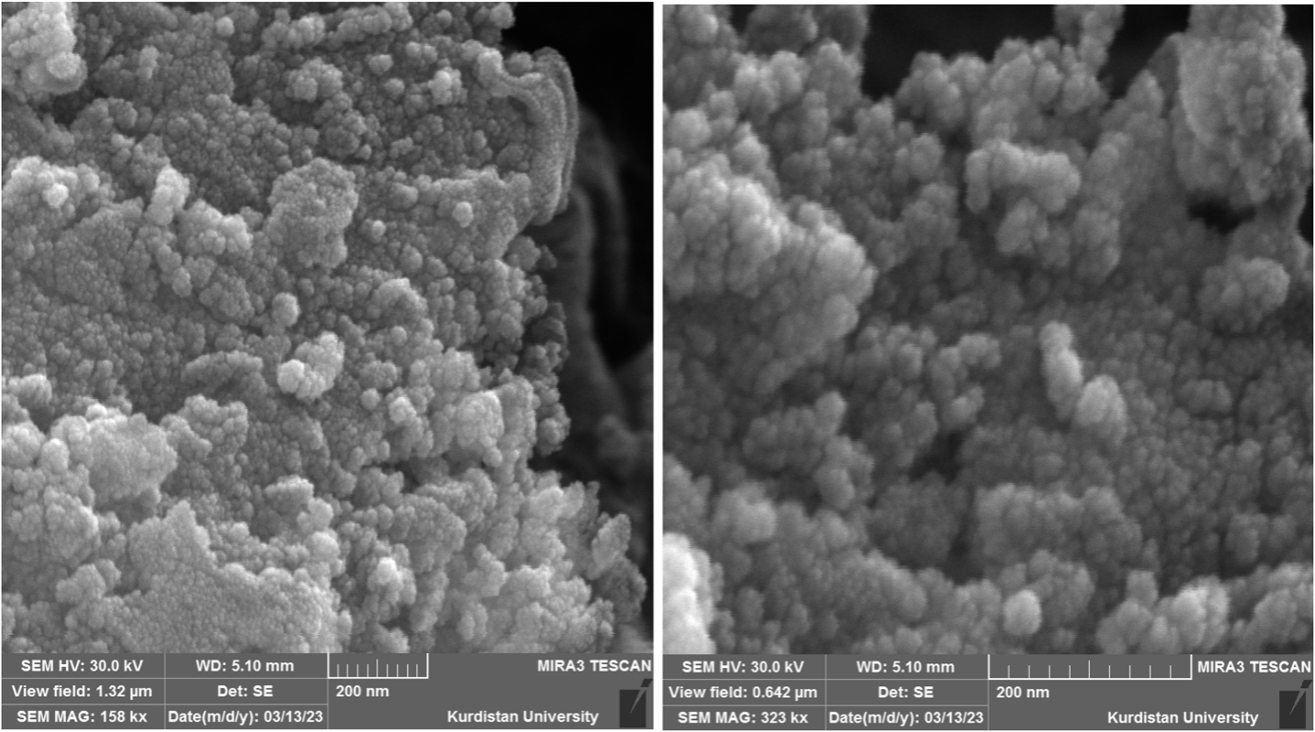
SEM images of Pd@boehmite.

**Fig. 5:**
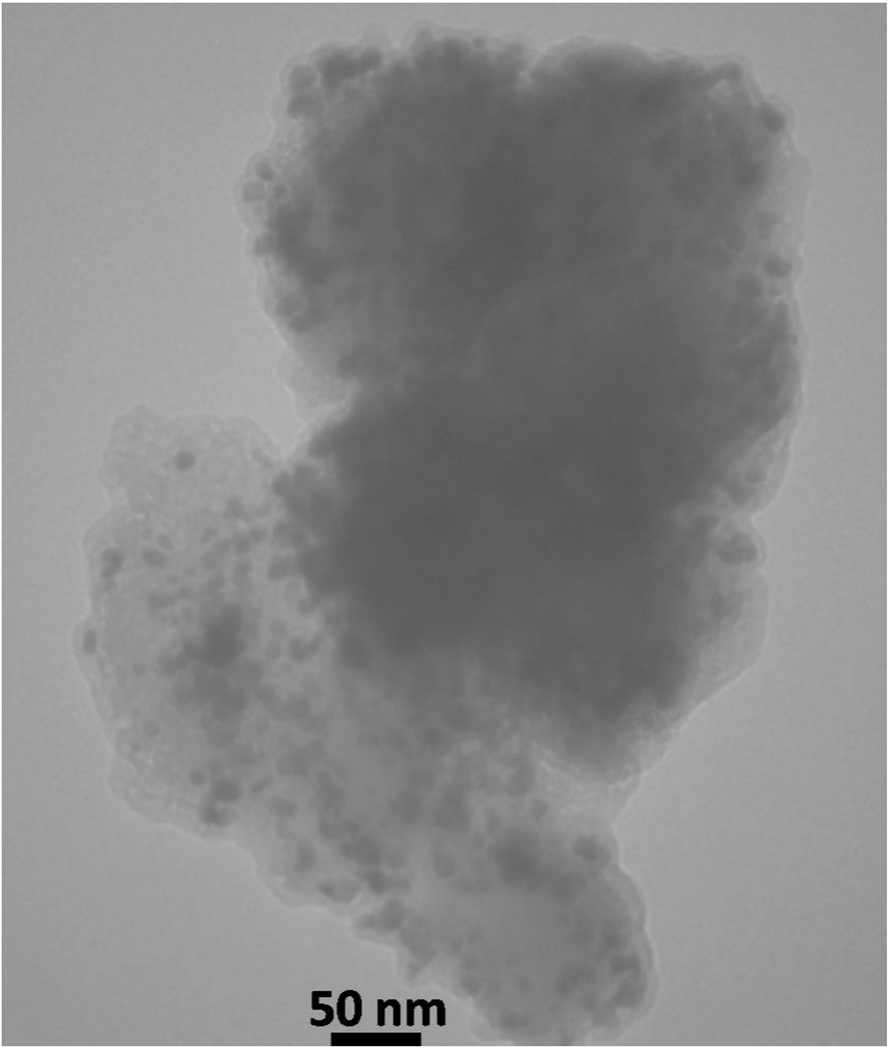
TEM images of Pd@boehmite.

The morphological and dimensional characteristics of Pd@boehmite were studied using a scanning electron microscope (SEM), and the images obtained being presented in Fig. 4. These images show a spherical morphology in nanometer dimensions for Pd@boehmite particles. Also, the TEM image of Pd@boehmite is shown in Fig. 5, which shows spherical palladium nanoparticles with dimensions less than 10 nm on boehmite.

### 3.2. Catalytic performance of Pd@boehmite

#### 3.2.1. Suzuki–Miyaura coupling reaction catalyzed with Pd@boehmite

The catalytic properties of Pd@boehmite were tested in the carbon–carbon bond formation *via* the Suzuki–Miyaura coupling reaction. The conditions of the Suzuki–Miyaura coupling reaction were investigated by changing various parameters (Table 1). First, the coupling of iodobenzene with phenylboronic acid was checked as a sample reaction without the presence of the Pd@boehmite catalyst, and the reaction did not occur after 6 h. Therefore, a catalyst is required for the Suzuki C–C bond formation. For this reason, different amounts of the Pd@boehmite catalyst were investigated in the coupling reaction of phenylboronic acid with iodobenzene (Table 1, entries 1–4), and 10 mg of Pd@boehmite was selected as the optimal amount of the catalyst (Table 1, entry 3). Then, we investigated the same reaction in different solvents (Table 1, entries 4–9), and finally, distilled H_2_O was selected as the best solvent for this described reaction. Finally, different types of inorganic and organic bases were tested in the same model reaction (Table 1, entries 9–11), and potassium carbonate was selected as the best base. Also, the required amount of base and the required temperature for the model reaction were investigated, and 1.5 mmol of potassium carbonate base and a temperature of 60 °C were considered optimal conditions.

**Table 1:** Optimizing conditions (various parameters) for the C–C bond formation in Suzuki–Miyaura coupling catalyzed by Pd@boehmite

Entry	Solvent	Pd@boehmite (mg)	Base type	Temp. (°C)	Time (min)	Yield^a^ (%)
1	H_2_O	—	K_2_CO_3_	80	360	N.R.^b^
2	H_2_O	8	K_2_CO_3_	80	20	95
3	H_2_O	10	K_2_CO_3_	80	15	96
4	H_2_O	15	K_2_CO_3_	80	10	96
5	PEG-400	10	K_2_CO_3_	80	45	92
6	1,4-Dioxane	10	K_2_CO_3_	80	420	55
7	DMF	10	K_2_CO_3_	80	420	61
8	DMSO	10	K_2_CO_3_	80	180	82
9	EtOH	10	K_2_CO_3_	80	420	70
10	H_2_O	10	NaOH	80	35	89
11	H_2_O	10	Et_3_N	80	30	72
12	H_2_O	10	K_2_CO_3_	60	30	91^c^

a Isolated yield using 0.75 mmol of base, aryl halide (0.5 mmol) and phenylboronic acid (0.5 mmol).

b No reaction.

c Isolated yield using 0.5 mmol of base.

Next, we synthesized various biphenyl derivatives using Pd@boehmite under optimized conditions (Table 2). In this step, aryl bromides and aryl iodides with electron-withdrawing or electron-donating substituents were successfully coupled with phenylboronic acid. All biphenyls were formed in good yields and in a short time. Also, aryl boronic acid derivatives with electron-withdrawing or electron-donating substituents were successfully coupled with aryl halides in the presence of Pd@boehmite. For example, the coupling of 3,4-difluorophenylboronic acid with aryl halides was used to synthesize the corresponding biphenyls in the presence of Pd@boehmite, and the biphenyl products were successfully synthesized. Also, 4-formylphenyl boronic acid and 4-methylphenyl boronic acid were coupled with iodobenzene in the presence of the Pd@boehmite catalyst and the corresponding products were successfully synthesized in good yields and in a short time (Table 2, entries 12 and 13).

**Table 2:** Synthesis of biphenyls through C–C bond formation in Suzuki–Miyaura coupling catalyzed by Pd@boehmite

Entry	Aryl halide	Aryl boronic acid	Biphenyl product^44,45^	Time (min)	Yield^a^ (%)
1	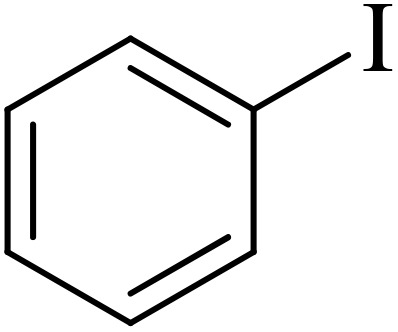	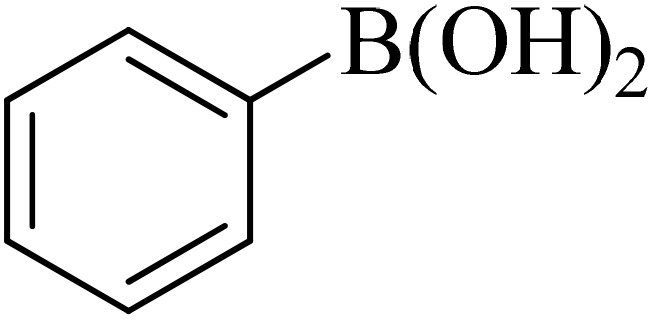	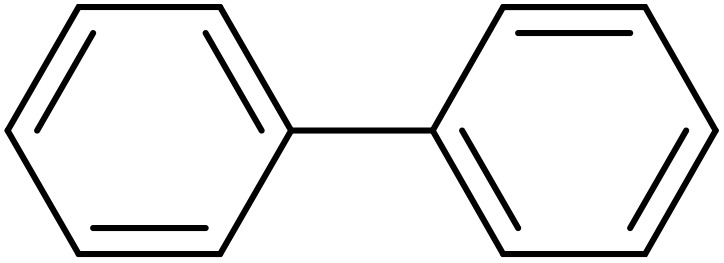	15	96
2	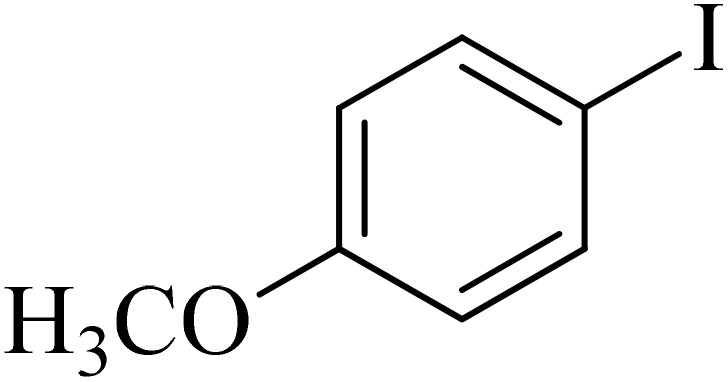	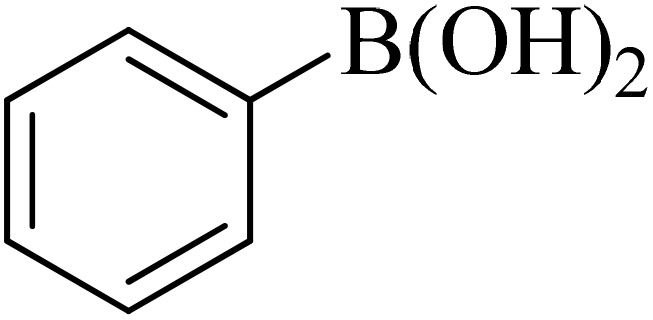	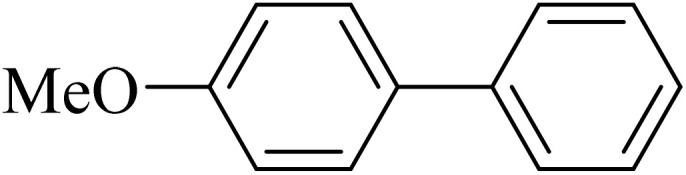	50	93
3	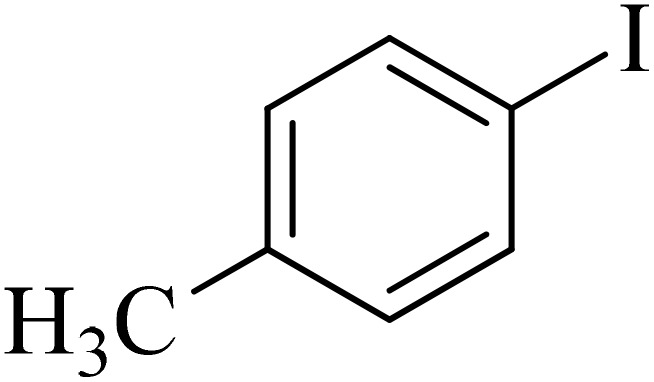	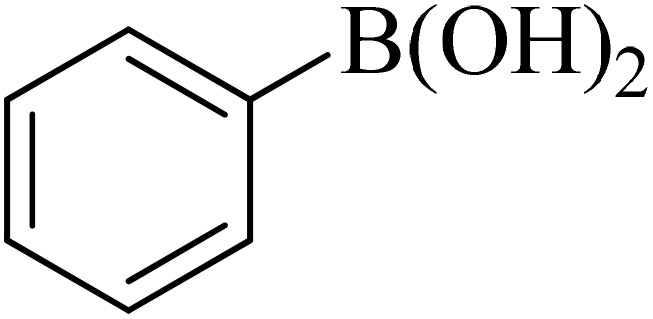	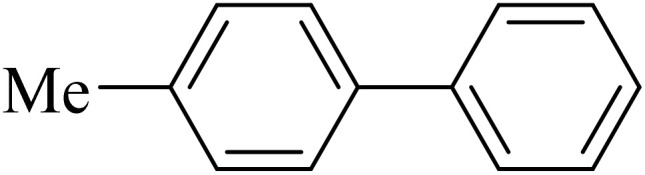	60	94
4	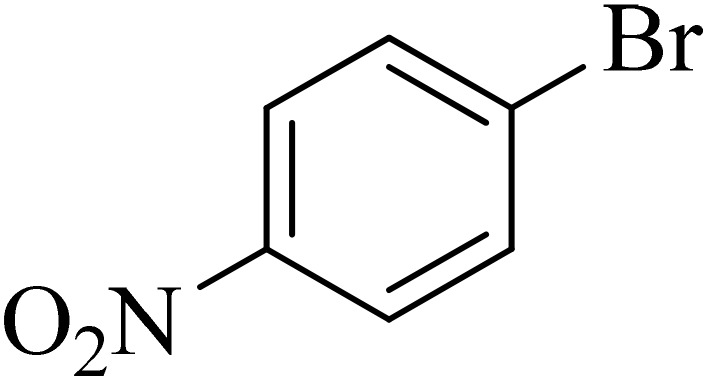	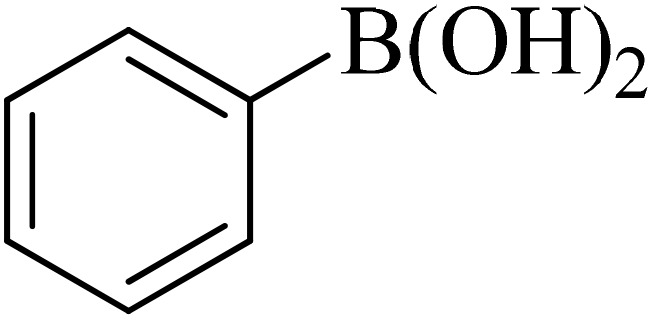	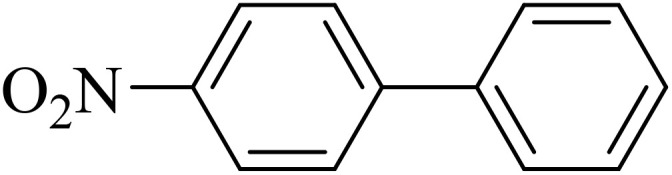	130	92
5	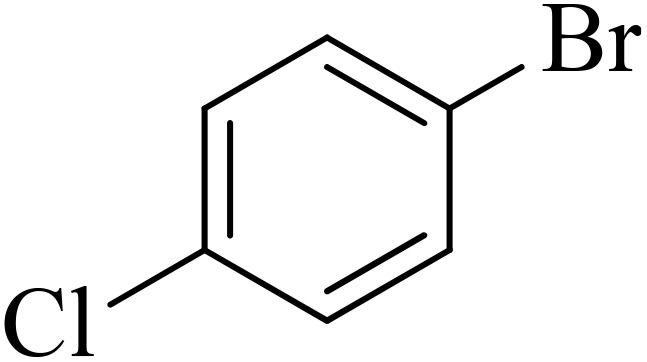	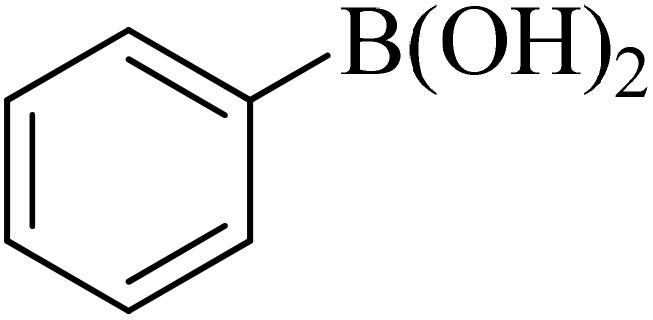	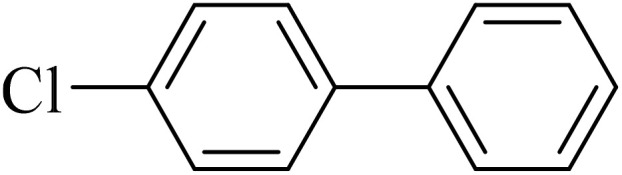	50	90
6	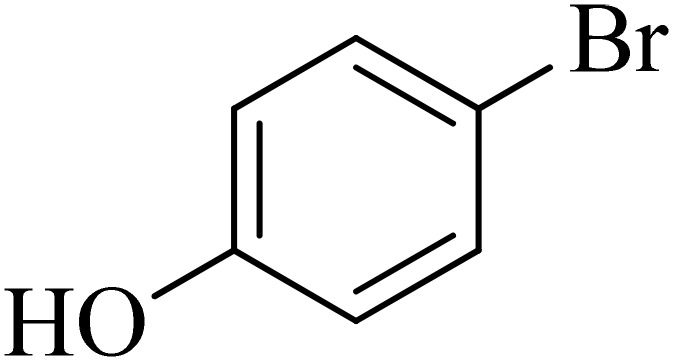	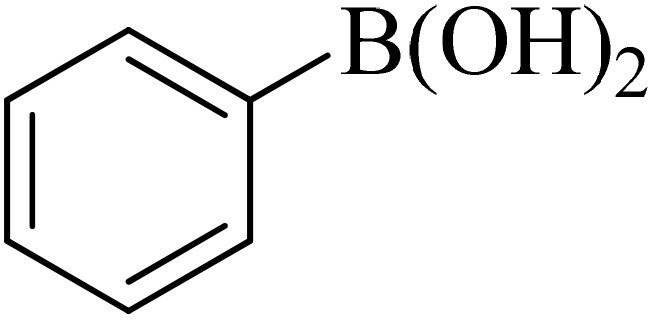	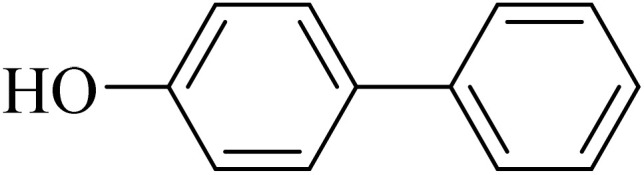	60	94
7	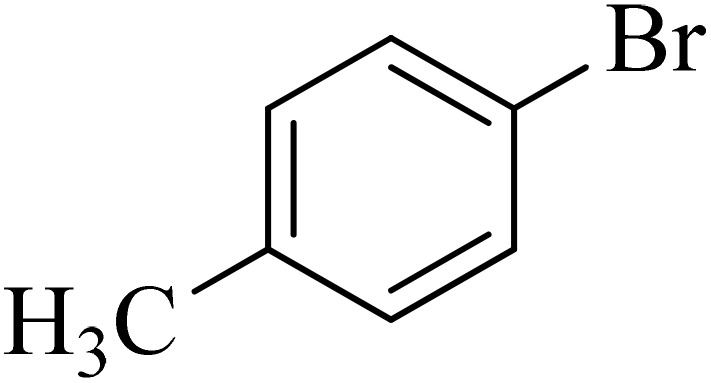	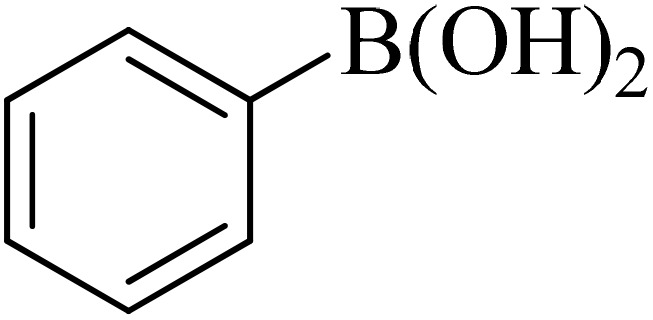	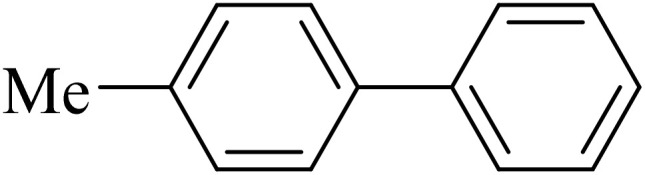	60	90
8	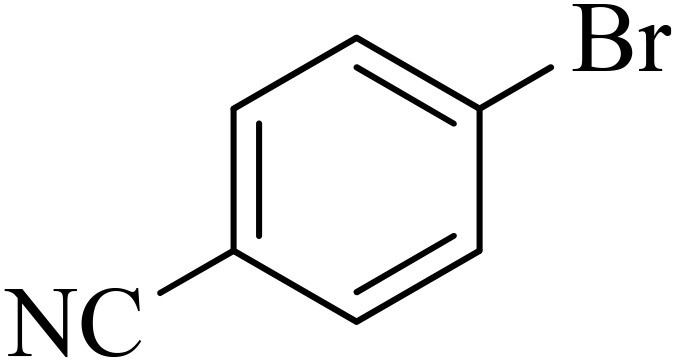	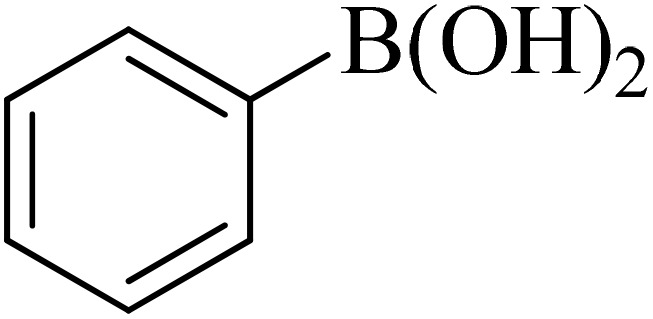	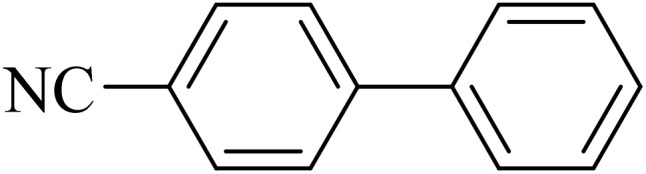	135	95
9	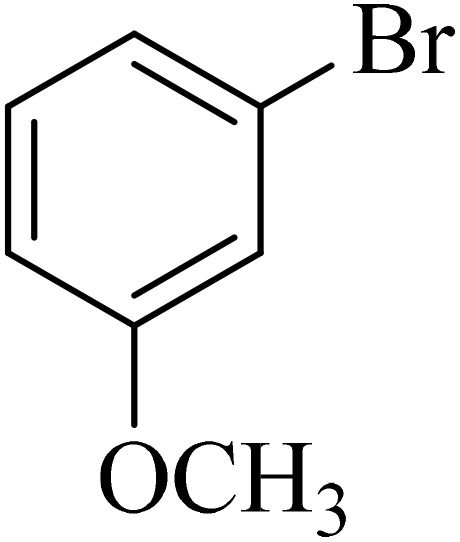	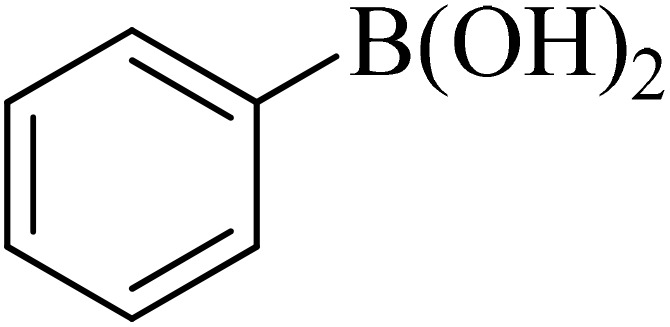	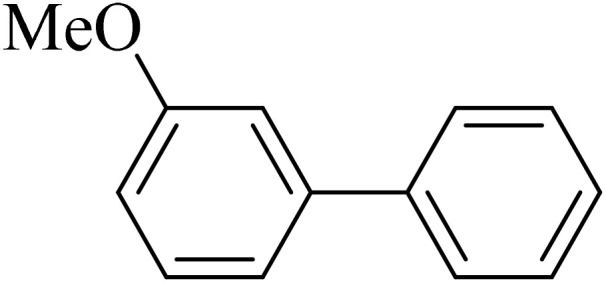	45	97
10	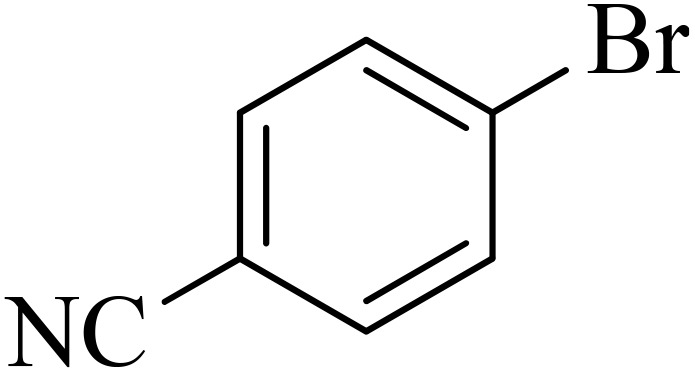	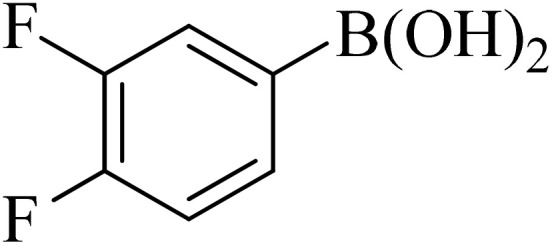	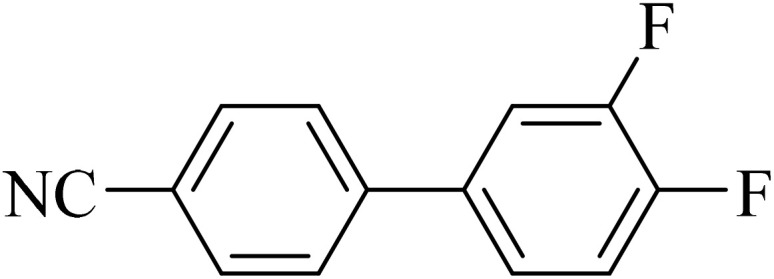	130	89
11	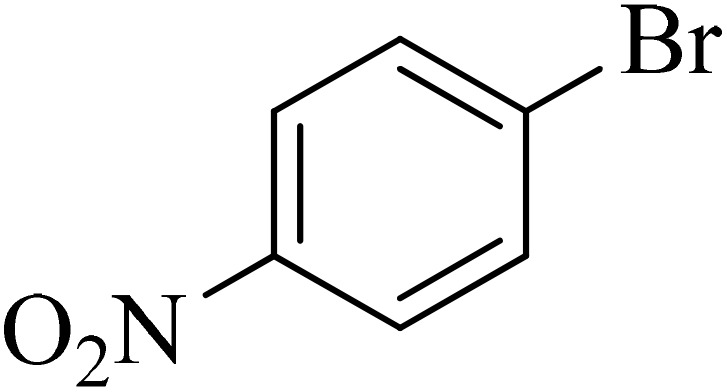	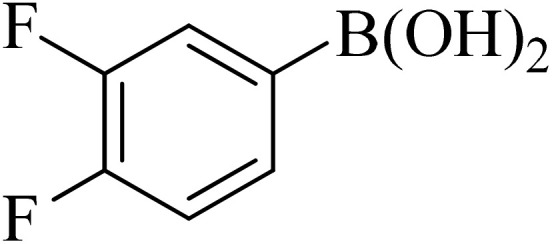	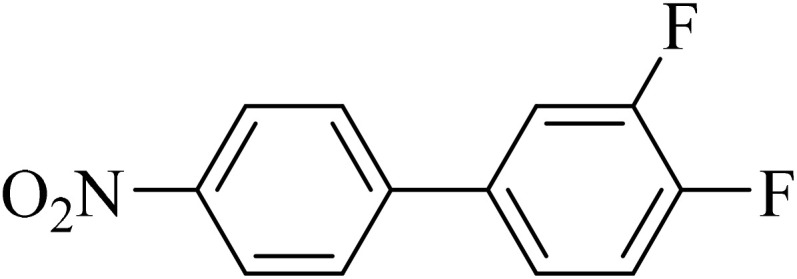	100	91
12	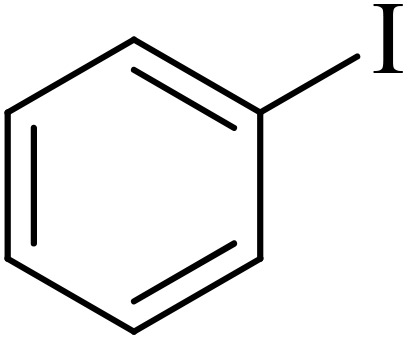	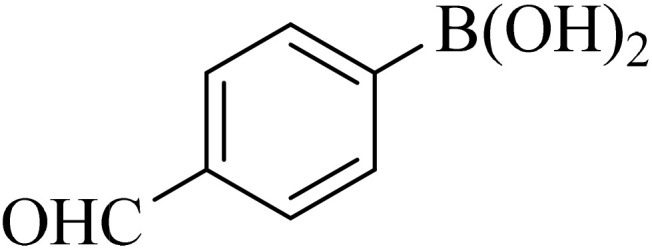	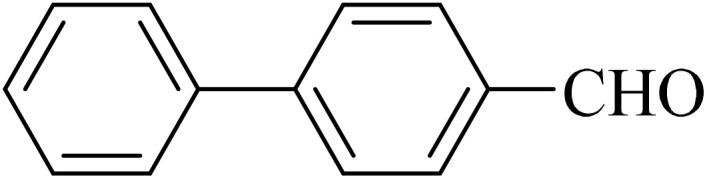	190	90
13	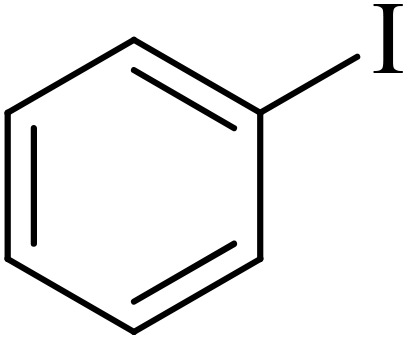	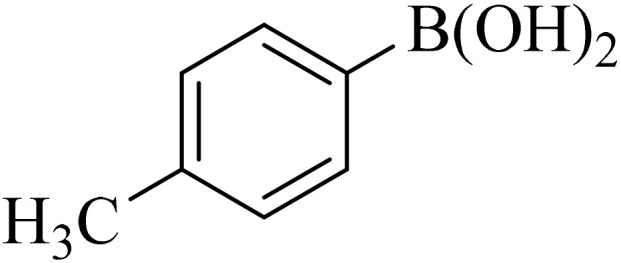	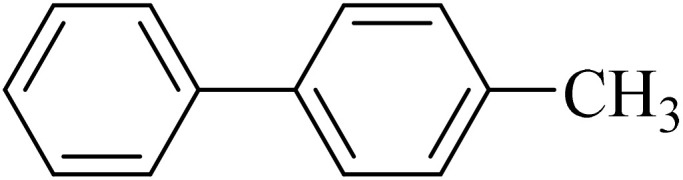	20	95

a Calculated yield.

**Scheme 3:**
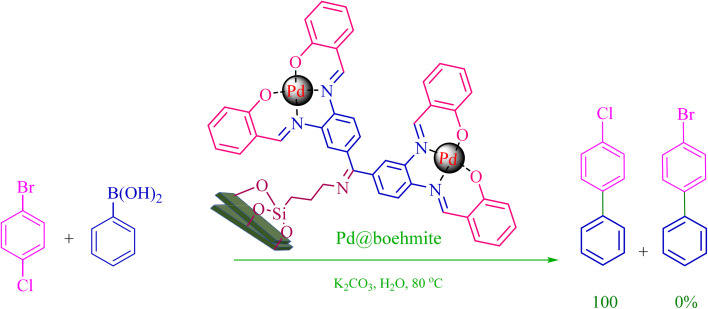
Selectivity in the C–C bond formation with the Pd@boehmite catalyst.

The Pd@boehmite catalyst showed good selectivity in the C–C coupling reaction. When the coupling of 1-bromo-4-chlorobenzene with phenylboronic acid was investigated (Table 2, entry 5), only its bromide moiety led to the formation of the C–C bond, and its chloride moiety remained selectively intact (Scheme 3).

**Scheme 4:**
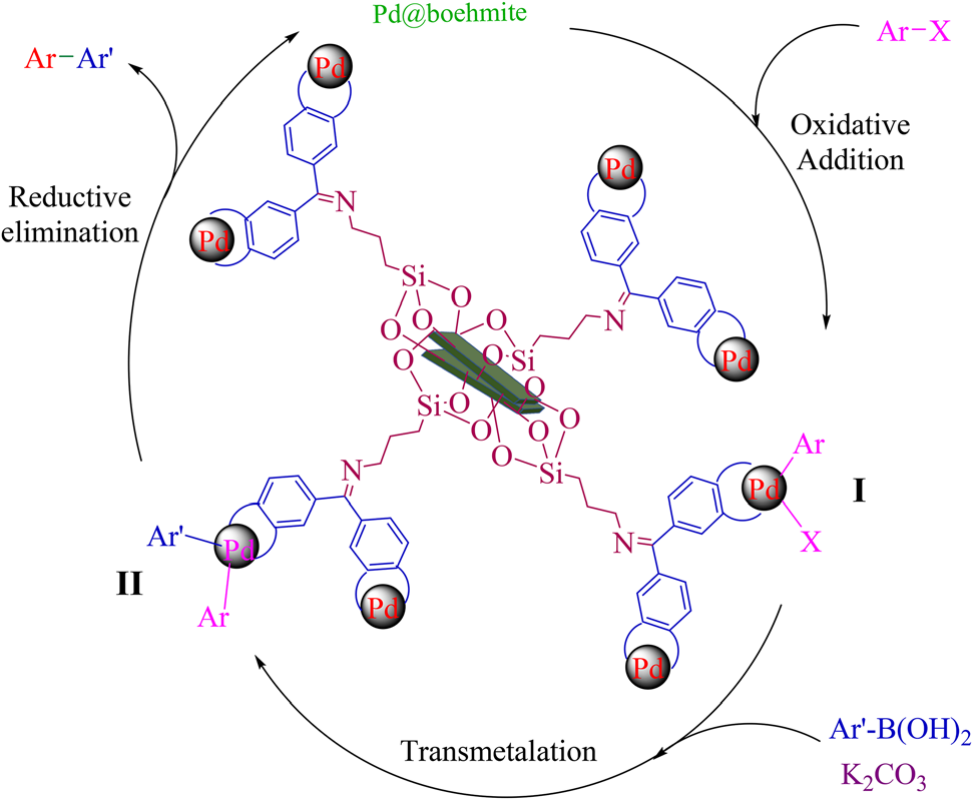
Suggested mechanism for the C–C bond formation catalyzed by Pd@boehmite.

Based on the history of the Suzuki–Miyaura coupling reaction in the literature,^46–52^ a mechanism for biphenyl formation by the Pd@boehmite catalyst is outlined in Scheme 4. The C–C bond formation in the Suzuki–Miyaura coupling reaction in the presence of a metal catalyst begins with an oxidative addition step in which palladium (0) is converted to palladium(ii) to form intermediate (I). Intermediate (I) is converted to intermediate (II) by a transmetalation process. Finally, intermediate (II) is converted to the product by a reductive elimination step, and palladium(ii) is converted back to palladium (0) and returns to the catalytic cycle.

#### 3.2.2. Reusability of the Pd@boehmite catalyst

**Fig. 6:**
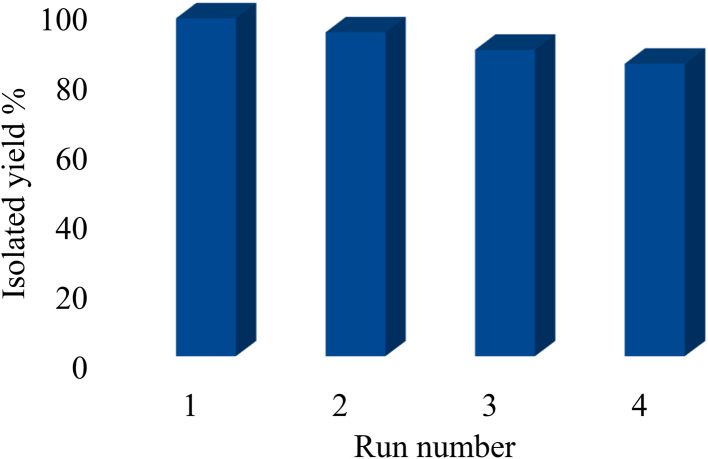
Pd@boehmite recycling in the synthesis of 3-methoxybiphenyl.

The recyclability of heterogeneous catalysts is their most important advantage. Therefore, heterogeneous catalysts are important in terms of green chemistry and economics. Thus, the recyclability of Pd@boehmite was investigated in the formation of C–C bonds under optimal conditions. For this purpose, the coupling reaction of 3-bromoanisole with phenylboronic acid in the presence of Pd@boehmite under optimal conditions was chosen as a model reaction. At the end of the reaction, Pd@boehmite was recovered and reused in the same reaction. As illustrated in Fig. 6, the Pd@boehmite catalyst can be recovered and reused in at least 4 steps.

#### 3.2.3. Comparison of the catalyst

Finally, the performance of the Pd@boehmite catalyst was compared with other catalysts in the literature. For this purpose, the results and conditions of the C–C coupling of iodobenzene with phenylboronic acid in the presence of the Pd@boehmite catalyst were compared with other catalysts in the literature (Table 3). All previous catalysts produced the 1,1′-biphenyl product in a long time, while the 1,1′-biphenyl product was synthesized using the Pd@boehmite catalyst after only 15 min. In addition, the product yield using the Pd@boehmite catalyst was higher than that of most old catalysts. In addition, many traditional methods used organic, toxic and expensive solvents to synthesize biphenyl derivatives, while the biphenyl derivatives were synthesized in the presence of the Pd@boehmite catalyst in an aqueous solvent, which is more suitable from the environmental, green chemistry and economic points of view. Also, the temperature required for the biphenyl formation using the Pd@boehmite catalyst is lower than that of most catalysts.

**Table 3:** Comparison results of Pd@boehmite in the formation of 1,1′-biphenyl through the coupling reaction of iodobenzene and phenylboronic acid with previously reported methods

Entry	Catalyst	Conditions	Time (min)	Yield (%) [ref.]
1	PANI–Pd	1,4-Dioxane : water (1 : 1), potassium carbonate, 95 °C	240	91 [53]
2	Pd(ii)–NHC complex	Dimethylformamide, cesium carbonate, 100 °C	24 h	99 [54]
3	Pd/Au NPs	Ethanol/water, potassium carbonate, 80 °C	24 h	88 [55]
4	Pd NP	Water, potassium hydroxide, 100 °C	12 h	95 [56]
5	Pd@SBA-15/ILDABCO	Water, potassium carbonate, 80 °C	90	97 [57]
6	Polymer anchored Pd(ii) Schiff-base complex	Dimethylformamide : water (1 : 1), potassium carbonate, 80 °C	300	99 [58]
7	CA/Pd (0)	Water, potassium carbonate, 100 °C	120	94 [59]
8	Ru–dithizone@biochar-Ni MNPs	Water, sodium carbonate, 80 °C	90	96 [60]
9	Pd(0)–TBA@biochar	Polyethylene glycol-400, sodium carbonate, 80 °C	125	96 [61]
10	Pd@boehmite	Water, potassium carbonate, 80 °C	15	96 [this work]

## 4. Conclusion

In conclusion, a new Schiff-base Pd-complex on functionalized γ-AlOOH (Pd@boehmite) was synthesized. At first, the γ-AlOOH surface was modified by 3-aminopropyltriethoxysilane and confirmed by UV spectroscopy, which indicated a strong peak at 575 nm. Then, it was functionalized with a Schiff-base ligand. Finally, the palladium complex was immobilized on its surface to form the Pd@boehmite catalyst. Pd@boehmite was characterized and its catalytic performance was investigated in the selective Suzuki–Miyaura coupling reaction. All biphenyl products were synthesized in excellent yields in distilled H_2_O solvent, which is safe, available, ideal, and environmentally friendly. Finally, the selectivity and reusability of the Pd@boehmite catalyst were investigated, which showed good selectivity and reusability.

## Conflicts of interest

There are no conflicts to declare.

## Supplementary Material

NA-007-D5NA00362H-s001

## Data Availability

All data generated or analyzed during this study are included in this published article and ESI.†
