# Supplementary material for: A new palladium complex Schiff-base on functionalized nanoboehmite as a reusable and practical catalyst for selective Suzuki C–C bond formation
Source: Nanoscale Adv. 2025 Jun 17;7(16):4867–75. doi: 10.1039/d5na00362h (PMC12208136; doi:10.1039/d5na00362h)

Supplementary Information (SI) for Nanoscale Advances.
This journal is © The Royal Society of Chemistry 2025

Supplementary Data

A new Palladium complex Schiff-base on functionalized nanoboehmite as a reusable and practical catalyst for selective Suzuki C-C bond formation

Samaneh Heydarian[1,*], Bahman Tahmasbi[2], Mitra Darabi[2]

[1] *Department of Chemistry, Dez.C., Islamic Azad University, Dezful, Iran, E-mail: Heydarian@iau.ac.ir*

[2] *Department of Chemistry, Faculty of Science, Ilam University, P. O. Box 69315516, Ilam, Iran, E-mail: b.tahmasbi@ilam.ac.ir*

Abstract

The surface of boehmite nanoparticles (γ-AlOOH NP) consists of hydroxy groups that enable its surface modification and functionalization. Based on this fact, we first functionalized the AlOOH NP surface with a Schiff-base ligand in this work. The Schiff-base ligand was synthesized from the reaction of *o*-formylphenol and (3,4-diaminophenyl)(phenyl)methanone. Then palladium nanoparticles were immobilized on it, which denoted as Pd@boehmite. Next, Pd@boehmite was investigated using TGA, DSC, SEM, TEM, and BET instrumental methods. Then, Pd@boehmite was used as a powerful catalyst for carbon-carbon bond formation in the Suzuki coupling reaction. Various aryl halide and aryl boronic acid derivatives were investigated using Pd@boehmite nanocatalyst and all biphenyl products were obtained with high yield and rapid reaction rate. Pd@boehmite showed good selectivity in synthesising biphenyls, when diaryl halide was used. Finally, the recyclability of Pd@boehmite was also examined, and this catalyst showed good reusability.

Keywords: Suzuki-Miyaura coupling, Pd-Schiff-base complex, boehmite, carbon-carbon bond formation, recyclable catalyst

^{1}H NMR spectral data

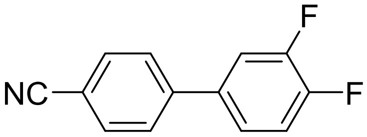

3',4'-difluoro-[1,1'-biphenyl]-4-carbonitrile

^{1}H NMR (250 MHz, $CDCl_3$): δ_H= 7.74 (d, J= 10 Hz, 2 H), 7.62 (d, J= 10 Hz, 2 H), 7.40 (t, J= 10 Hz, 1 H), 7.30 (m, 1 H), 7.26 (m, 1 H) ppm.

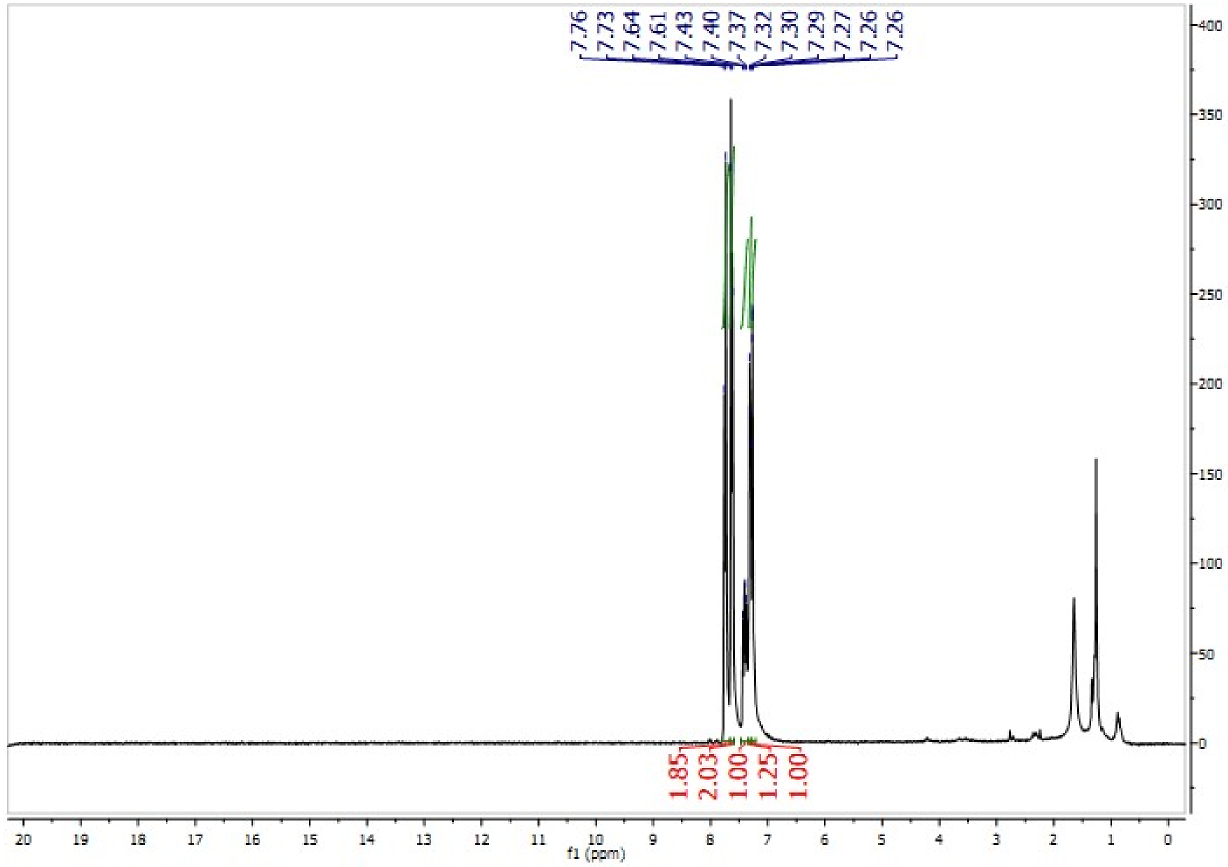

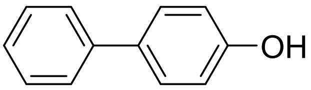

[1,1'-biphenyl]-4-ol

^{1}H NMR (250 MHz, $CDCl_3$): δ_H=7.55(d, *J*= 10 Hz, 2 H), 7.52(d, *J*= 10 Hz, 2 H), 7.42(t, *J*= 6 Hz, 2 H), 7.31(t, *J*= 6 Hz, 1 H), 6.91(d, *J*= 10 Hz, 2 H), 4.26 (br, 1 H)ppm.

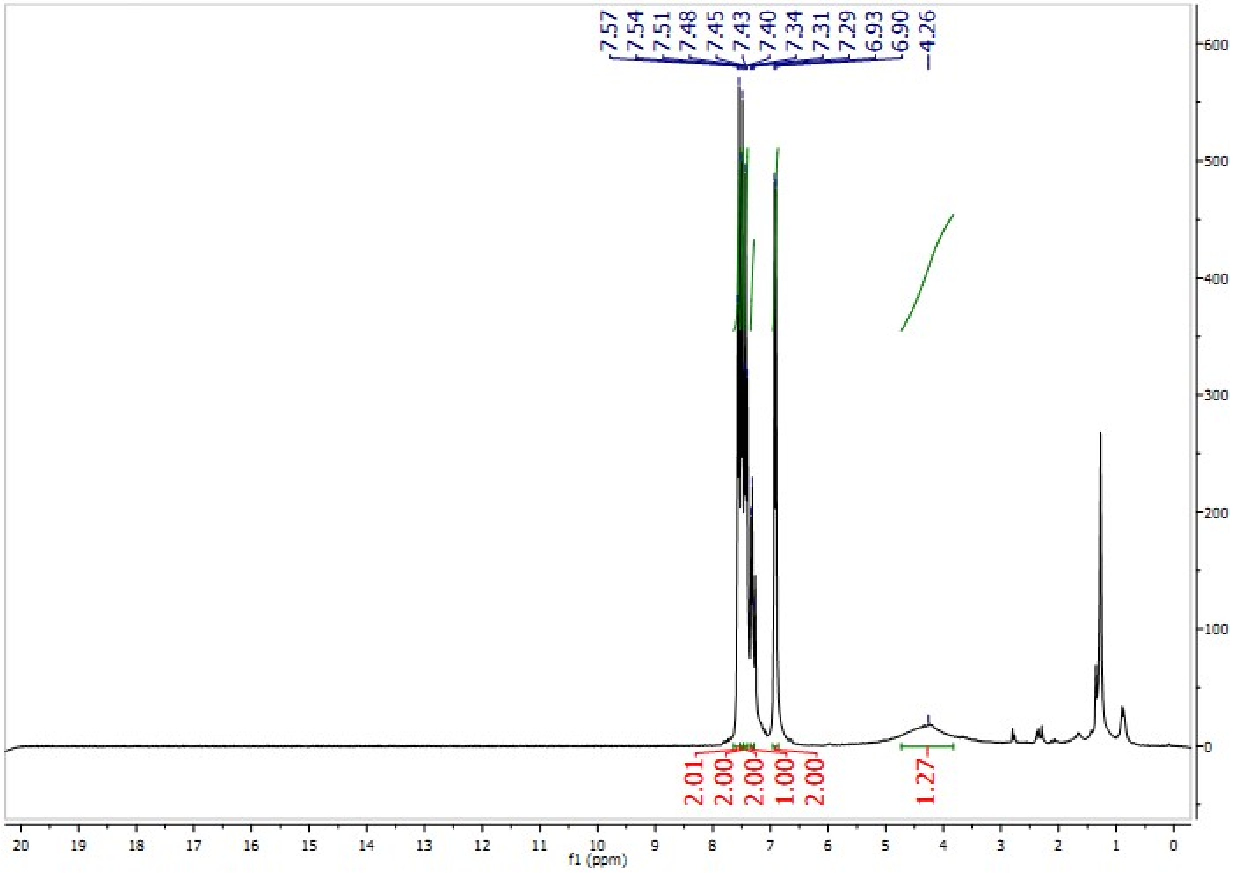

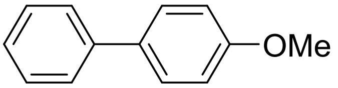

4-methoxy-1,1'-biphenyl

^{1}H NMR (250 MHz, $CDCl_3$): δ_H= 7.54 (m, 4 H), 7.42 (t, *J*= 10 Hz, 2 H), 7.25 (m, 1 H), 6.98 (d, *J*= 5 Hz, 2 H), 3.86 (s, 3H) ppm.

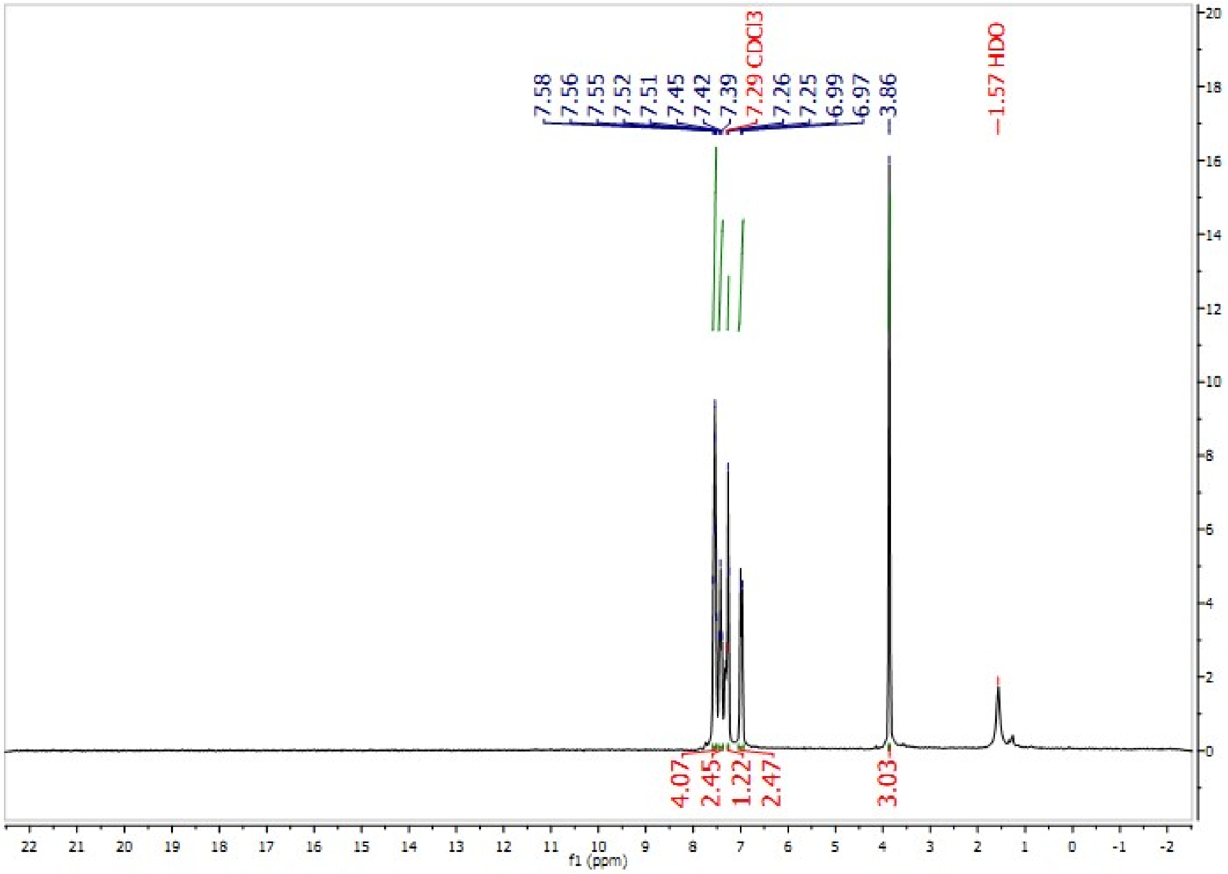

Supplement: NA-007-D5NA00362H-s001 [file NA-007-D5NA00362H-s001.pdf]
